# Editorial: The intersection of genes, nutrition and disease: a nutritional perspective from Mendelian randomization to disease pathogenic mechanisms

**DOI:** 10.3389/fnut.2025.1631608

**Published:** 2025-06-03

**Authors:** Chaoyan Yue

**Affiliations:** Department of Clinical Laboratory, Obstetrics and Gynecology Hospital of Fudan University, Shanghai, China

**Keywords:** genes, nutrition, disease, precision, Mendelian randomization

In the wave of medical revolution in the 21st century, nutrition science is undergoing an unprecedented paradigm shift. Based on the breakthrough development of genome-wide association studies (GWAS) technology and the innovative application of Mendelian randomization (MR) methods, researchers have been able to reinterpret the classic scientific proposition of “nutrition health disease” from a genomic perspective.

This Research Topic focuses on the intersection of “genes, nutrition, and disease,” systematically integrating multidisciplinary research findings to provide an innovative perspective for revealing the three-dimensional interaction network of nutrition-genes-disease and building a personalized nutritional intervention system. Key findings include: the host-genetic-driven habit of adding salt to food may significantly elevate the risk of delirium, while habits such as using low-fat polyunsaturated margarine in cooking, consuming cheese, and drinking coffee appear to mitigate delirium risk. Elevated levels of genetically predicted 1-stearoylglycerol and 3-methoxytyrosine may contribute to reducing delirium susceptibility, suggesting a protective role against delirium development. These insights into the potential causal associations between dietary components and blood metabolites with delirium risk point toward targeted dietary intervention strategies and the development of novel biomarkers (Zhu et al.). Elevated serum uric acid (SUA) levels significantly increase the risk of cervical adenocarcinoma. A positive correlation was also observed between SUA levels and cervical squamous cell carcinoma, although this association did not reach statistical significance. These results emphasize the importance of monitoring and managing elevated SUA levels in cervical cancer prevention strategies, particularly for high-risk female populations. Furthermore, our study supports incorporating SUA levels into cervical cancer risk prediction models, which could help improve public health guidelines and nutritional interventions (Cao et al.). Specific causal relationships between trace elements, nutrients, and diabetes subtypes along with their complications provide critical evidence for precision prevention and treatment strategies. Using published GWAS data, the study inferred causal relationships between serum trace elements and vitamins with different subtypes of diabetes and its complications. The analysis revealed that selenium is associated with an increased risk of type 2 diabetes; vitamin B6 is associated with an increased risk of neurological complications in type 2 diabetes; magnesium is negatively related to the risk of developing type 1 diabetes; carotene is associated with an increased risk of kidney complications in type 1 diabetes; vitamin B12 is negatively related to the risk of kidney complications in type 1 diabetes; carotene is associated with an increased risk of neurological complications in type 1 diabetes; potassium and vitamin B6 are negatively related to the risk of neurological complications in type 1 diabetes; vitamin E is negatively associated with the risk of peripheral circulatory complications in type 2 diabetes. Multivariable Mendelian randomization analysis showed that vitamin B6 affects type 1 diabetes with neurological complications and type 2 diabetes with neurological complications independently of other exposure factors, vitamin B6 may affect type 1 diabetes with renal complications independently of other exposure factors, and vitamin E may affect type 1 diabetes with peripheral circulatory complications independently of other exposure factors (Jia and Chen). Additionally, the study confirmed that increased values of genetically predicted obesity-related anthropometric indicators, including BMI, waist and hip circumferences, basal metabolic rate, body fat percentage, whole-body fat mass, trunk fat mass, arm fat mass, leg fat mass, whole-body fat-free mass, trunk fat-free mass, arm fat-free mass, leg fat-free mass, and whole-body water mass, can increase sepsis risk, providing a scientific basis for screening high-risk populations in clinical and community settings (Zhang et al.). The negative association between green tea consumption and male attention deficit hyperactivity disorder (ADHD) reveals the potential value of dietary interventions in the prevention and control of neurodevelopmental disorders (Chen et al.). Together, these groundbreaking discoveries advance nutritional medicine from “macro-dietary recommendations” to “micro-metabolic regulation.”

The research in this Research Topic breaks through the limitations of traditional observational studies by systematically applying Mendelian randomization and genome-wide association studies, revealing for the first time the relationship between nutrition, genes, and diseases in the causal dimension. This not only echoes the urgent need to reduce disease risk through dietary management in the context of the increasing global burden of chronic diseases, but also lays the methodological foundation for the transition of “precision nutrition” from concept to practice. Future research can further combine genetic data with functional experiments to achieve breakthroughs in causal mechanism analysis and intervention plan optimization, truly promoting nutrition from “risk association discovery” to “precise intervention implementation.” Nutritional science will play an increasingly important role in global health governance. We look forward to more researchers focusing on the interdisciplinary field of “genetics, nutrition, and disease” to jointly promote the deep integration of nutrition science and precision medicine, and contribute more wisdom and strength to maintaining human health and addressing global health challenges.

